# Understanding the Relationship Between Decreases in Social Security Benefits and Intergenerational Inequalities in Mental Health

**DOI:** 10.1177/27551938231185948

**Published:** 2023-07-04

**Authors:** Julija Simpson, Clare Bambra, Heather Brown

**Affiliations:** 1 Population Health Sciences Institute, 12186 Newcastle University, Newcastle upon Tyne, UK; 2 Department of Health Research, 4396 Lancaster University, Lancaster, UK

**Keywords:** mental health, welfare reform, adolescents, intergenerational inequalities

## Abstract

It is well-established that mental health follows similar patterns across generations. However, little is known how structural factors, such as those related to social security reforms, may impact this relationship. Our aim was to quantify the strength of association in mental health between parents and their adolescent children, and to explore how much of this correlation is explained by decreases in benefits. We used data from U.K. Household Longitudinal Study (2009–2019) from which we matched youth data to their parents, and split the sample into single- and dual-parent households. To estimate the intergenerational correlations, we estimated a series of unit- and rank-based regression models of standardized and time-averaged mental health measures for adolescents and their parents. Our findings suggest that there are statistically significant intergenerational associations in mental health between parents and children for both single- and dual-parent households, with the relationship being stronger for single-mother households. Benefit losses explain a small proportion of this association, for both single-mother and dual-parent households. Nevertheless, they are negatively associated with the mental health of adolescents in dual-parent households—independently of both adolescent and parental characteristics. Such negative effects should be considered when designing and evaluating future social security benefit policies.

## Introduction

### Background

Over the past decade, the U.K. social security system has undergone a series of extensive welfare reforms, broadly characterized by cuts in benefit eligibility and generosity. The key benefit reforms were legislated via two acts—the Welfare Reform Act 2012 and Welfare Reform and Work Act 2016—and included contractionary policy measures such as lower benefit uprating, increased work requirements and imposition of a household benefit cap, or a cap on the total benefits a household can receive (for more detail on the U.K. welfare reforms, see Hobson).^1^ Although the key stated aim of these reforms was to incentivize the transition from welfare to work,^2^ some scholars have argued that the real aim at least of some of the reforms (e.g., increased conditionality) was to punish and control benefit claimants through increasing social and material losses,^3^ or via “criminalization of poverty”.^4^

Indeed, families that were most affected by the reforms experienced significant financial losses. For example, the poorest 10 percent of families who rely on benefits as their main source of income lost 20 percent of their income on average as a direct result of these reforms.^5^ Further evidence also suggests that benefit income fell more sharply among families with no earners^6^ and, among those with children, single-parent households were particularly affected.^7–9^ Furthermore, although there were significant increases in employment of single parents during the reform period (2009–2019),^10^ there is evidence to suggest that the accompanying increases in labor income did not offset benefit losses, leaving single-parent families worse off on average than prior to the reforms.^11^

Emerging public health literature shows that the reforms had negative impacts on the mental health of those affected, including single parents, the unemployed, and those with disabilities.^12–15^ However, while concerns have been raised about the potential impacts of these reforms on children,^16^ to date, little is known about their potential intergenerational effects.

There is a growing body of evidence suggesting that mental health is correlated across generations. Children whose parents have poor mental health tend to have worse mental health themselves, both in childhood/adolescence as well as later in adulthood.^17,18^ While a part of this correlation could be explained by genetic factors and shared household environment, or interaction between the two,^19,20^ it is possible that, by influencing the shared household environment, broader economic factors, such as those related to social security reforms, can also contribute to the intergenerational correlation in mental health. For example, international comparisons suggest that countries with more extensive welfare state provision and universal health care systems, such as those in Nordic countries, tend to have higher levels of health mobility (i.e., less intergenerational persistence) compared to countries like the United States that have less extensive public service provision.^21^ Nevertheless, thus far, there have been very few studies focusing on exploring intergenerational correlations in mental health and the potential drivers of this relationship. Two notable exceptions include the studies by Brown^22^ and Vera-Toscano and Brown.^23^

The study by Vera-Toscano and Brown^23^ has found a significant intergenerational correlation for mental health between adults and their young adult children (aged 25–35) in Australia, estimated between 0.18–0.21, depending on the inclusion of covariates. This means that between 18–21 percent of mental health of the parents is transmitted to their children—a finding common in the literature in this field.^18–24^ The study has additionally shown that early life disadvantage is an important factor influencing the intergenerational correlation in mental health, providing evidence on the importance of contextual factors in reducing intergenerational health disadvantage and inequalities.

The study by Brown^22^ investigated the role of changing policy focus in the United Kingdom on the intergenerational transmission of wages, self-assessed health, and mental health. The study utilized 18 waves of data covering the period 1991–2017 from the British Household Panel Survey and its successor Understanding Society Survey. To investigate the role of policy, the study divided the available timeframe into three distinct policy periods: 1991–1998 (increasing neoliberalism), 1999–2009 (English Health Inequalities Strategy), and 2010–2017 (Austerity). The study has found that for the population on average, changing policy focus had no impact on the strength of the relationship across generations in both health and mental health and wages. The study has additionally explored the role of policy environment on inequalities by parental marital status. Although no significant subgroup differences were found for mental health, when looking at self-assessed health and wages, the study found a slight weakening in the influence of parents on their young adult children in single- but not dual-parent households, indicating that the policy environment may have differential effects on the influence of the family on health depending upon parents’ marital status and can therefore impact inequalities.

In this study, we explore the role of the recent U.K. social security reforms as a potential driver of intergenerational persistence in mental health and mental health inequalities between parents and their adolescent children (aged 10–15). To operationalize the role of social security reforms, we focus on the effects of benefit decreases—a key economic mechanism potentially linking social security reform and mental health, as discussed in more detail below.

### Potential Mechanisms

Social security reforms can affect intergenerational transmission of mental health by affecting the shared household environment of both parents and children. One key mechanism via which social security reforms can affect parents is by inducing changes to benefit income.^25^ The pathways via which social security reforms can in turn affect children depend on both parental behaviors and their well-being. Two central theories focusing on parental economic characteristics could help explain the effects of social security benefit changes on children: the “Investment” model^26,27^ and the “Parental Stress” model.^28^

The “Investment” model focuses on the time and monetary investments of parents to their children and their potential effects on child well-being. Based on this theory, cuts in benefits would reduce monetary investments in children and likely have negative effects on their well-being, particularly in single-parent households that rely on a single source of income. The “Parental Stress” theory, on the other hand, suggests that, by affecting material living standards, benefit cuts can impact the level of stress of the parents, which in turn affects their parenting and therefore indirectly affects the mental health of their children. Based on this latter theory, we would expect benefit losses to help explain a non-negligible proportion of the intergenerational correlation in mental health.

### Aims

There have been increasing concerns about the effects of recent social security benefit cuts in the United Kingdom on the mental health of both parents and children, particularly those in single-parent families.^29^ While there is evidence on the association between child mental health and social and economic outcomes in adulthood,^30^ there is a gap in the literature on how social security policy may be exacerbating existing inequalities in mental health.

The aim of this study is therefore to contribute to the literature on social security benefit changes and the intergenerational transmission of health inequalities.

The specific objectives are to:

1. Estimate the intergenerational correlation between parental and adolescent (aged 10–15) mental health;
2. Estimate the impact of household benefit losses on the intergenerational correlation in mental health; and
3. Compare the correlations in mental health between single- and dual-parent households in order to investigate the role of social security benefit changes on intergenerational mental health inequalities.

By exploring the role of benefit losses on the intergenerational correlation, our ultimate aim is to provide evidence on potential areas of social policy interventions to improve mental health mobility and reduce mental health inequalities.

## Methods

### Data

The data source of this analysis was the U.K. Household Longitudinal Study (UKHLS),^31^ covering the period between 2009 and 2019 (i.e., waves 1–10). UKHLS is a large, nationally representative, longitudinal panel survey based on a stratified clustered random sample of 40,000 households from the four U.K. countries. Sample selection for the survey is based upon postcodes, which are then grouped into geographical strata to ensure a nationally representative selection of households. The survey asks respondents a range of questions related to their health, labor market experience, finances, opinions, family life, and well-being. For more detail on the survey design, see Jackle and colleagues.^32^

To derive the analysis sample, we first selected all observations of eligible mothers (aged 16–65) and merged the available paternal data for partnered mothers using the core questionnaire of the survey. We then merged the adult data with the youth sample data (derived from the youth questionnaire, administered to adolescents aged 10–15), keeping all available waves from the parental survey (for some of which youth data were not available). This is because we aimed to maximize the available data for benefit losses, the calculation of which required both current (t) and previous wave (t-1) observations. This meant that even though some of the youth data for time t-1 might be missing, we were still able to calculate the benefit losses if their parental data were available. In addition, given the focus on the effect of benefit losses (and not gains), only individuals experiencing benefit losses were kept in all analyses.

The analysis was carried out separately for single-mother^a^ and dual-parent households. We defined a mother as “single” if she described her relationship status as either unmarried, separated, divorced, or widowed. For defining dual-parent households, we included mothers who described their relationship status as either married or cohabiting and included only those whose partners’ data were non-missing. We further restricted our sample to parents who maintained their partnership status at both t-1 and t (previous and current wave, respectively). This reduced the possibility that the results could be driven by changes in partnership status, a potentially endogenous variable between social security reform and adolescent mental health.

The total number of observations available was 5,220 for single mothers and 21,321 for dual parents, yielding a total available sample size equal to 26,541 observations. As noted, the key youth outcome data, however, was only available in odd waves, leaving approximately 1,340 and 4,200 observations available to use in the regression analyses for single-mother and dual-parent households, respectively, depending on the covariates included.

### Outcomes

The main outcome of interest in this analysis was the correlation in mental health between adults and their adolescent children. Adult mental health was measured by the 12-item General Health Questionnaire (GHQ-12). Adolescent mental health was measured by the Strength and Difficulties Questionnaire (SDQ).

It should be noted that, ideally, we would have used two identical measures of mental health for parents and their offspring. This is the standard practice in the literature on intergenerational correlations, though there are exceptions.^b^ However, given that our analysis focuses on parents and children at different points in their lifecycle (i.e., adulthood and adolescence), there were no identical measures of mental health available in the survey. This is because GHQ-12 (which is one of the main mental health measures in UKHLS and other surveys) has been designed to detect psychiatric morbidity in adult populations.^34^ It is therefore not usually administered to children or adolescents, for whom the SDQ measure is typically used. Nevertheless, both GHQ-12 and SDQ are validated mental health measures and both capture aspects of mental health, including concentration, worry, happiness, depression, and confidence (see Supplementary Material [Table 1S] for a side-to-side comparison of the two measures). Therefore, the two measures were deemed to be sufficiently similar to be included in the analysis.

For ease of interpretation and comparability with other studies, the mental health measures for both adults and children (i.e., SDQ and GHQ-12) were standardized with mean 0 and standard deviation 1 and reverse coded, with higher scores indicating better mental health.

### Key Explanatory Variable – Benefit Loss

Our main explanatory variable is time-averaged monthly household benefit loss (in £). The benefit loss measure was calculated by subtracting the benefit income from the current wave (t) from the previous wave (t-1), keeping only observations with losses (and not gains). The benefit loss measure was logged, equivalized, and deflated to 2015 prices. It should be noted that for the analysis of dual-parent households, we used the average values of both parents for all explanatory variables to capture the key information of both parents. This is a standard procedure commonly used in the literature on intergenerational correlations in mental health (e.g., Vera-Toscano and Brown).^23^

### Other Covariates

Following previous literature (e.g., Johnston and colleagues),^18^ we included control variables for a number of additional observable characteristics that could explain the intergenerational correlation in mental health. These included: adolescent age, age squared, and sex (1 = Female; 0 = Male), ethnicity (1 = Non-White; 0 = White); parental age, age squared, and sex (1 = Female; 0 = Male); parental educational attainment (1 = No degree; 0 = With degree); number of children in the household; and region. In line with the previous literature, the time-varying control variables were time-averaged for each parent and child. More detailed definitions of the variables used in the analysis are presented in Supplementary Material (Table 2S).

### Econometric Framework

Our first objective was to explore whether there is an association between parental and adolescent mental health, for single- and partnered-parent households separately. We investigated this by running linear regression models, estimated by Ordinary Least Squares, with standard errors clustered at household level in order to account for the fact that some families have multiple children. Specifically, we estimated models of the following form:

(1) $$y_{it}^{C}=\;\alpha_{0}+\beta_{1}y_{it}^{\;p}+\beta_{2}X_{it}+\varepsilon_{it}$$

where $\alpha_{0}$ is the intercept; $y_{it}^{C}$ and $y_{it}^{p}$ are child and parent mental health status, respectively; $X_{it}\;$ is the vector of adolescent and parent control variables, including adolescent sex, age, age squared, and ethnicity, with parental variables including age, age squared, education, and the number of children in the household; and $\varepsilon_{i}$ is the random error term. The coefficient of interest is $\beta_{1}$ , representing the estimate of intergenerational association in mental health, with *1*- $\beta_{1}$ representing the degree of intergenerational mobility. All models were estimated using Stata v.16.^35^

Our second objective was to investigate how much of the correlation in mental health across the two generations could be explained by average monthly household benefit losses. Following an approach commonly used in the literature in this field, we explored the impact of this factor by adding it to the main regression models using a stepwise process (i.e., by adding benefit losses to the fully adjusted model specification and comparing the results to then calculate the proportion explained by benefit losses).

The literature on intergenerational mobility has emphasized two inherent biases in estimating the degree of persistence in a society: attenuation bias and lifecycle bias. Attenuation bias reflects a bias arising from measurement error in only including, say, single-year measurements to capture lifetime health. This means that there will be substantial noise in such estimates, leading to an attenuation of the estimated parameters. In the income mobility literature, attenuation bias has been regarded as one of the key empirical issues affecting the findings.^36^ To reduce this bias, the consensus in the literature is to use time average values of health for both generations. Halliday and colleagues^37^ suggest that reliable estimates of intergenerational correlation can be obtained by using approximately four to five years of health status for the parents. We followed the recommended approach and calculated lifetime averages for both parents and their adolescent children.

Lifecycle bias captures biases arising from measuring outcomes at suboptimal ages—that is, at certain points in their lifecycle that do not accurately proxy their lifetime outcome.^38^ To overcome this bias, it is suggested to evaluate the mental health of both generations at lifecycle points as close as possible (i.e., similar ages of parents and their offspring).

Another potential issue related to measurement is that it is possible that the health distribution becomes more/less compressed in the child distribution than it was in the parent generation, suggesting that rank-based measures of correlation might more accurately reflect the degree of intergenerational persistence than linear unit-based associations.^39,40^ For this reason, in addition to linear intergenerational health associations, following the approach by Chetty and colleagues,^40^ we also estimated rank-based coefficients by first calculating percentile ranks of mental health measures in each generation, and then estimating regressions as described above but for ranks ranging from 1–100 percentiles. If there are no differences in distribution between parental and adolescent measures, the rank-based coefficients and intergenerational health associations should provide the same results. Halliday and colleagues^37^ have shown that rank-based measures are also more robust to measurement error. In the interpretation of the findings, we therefore prioritized the rank-based estimates to those that are unit-based.

We also investigated the impact of item non-response on the main results. First, we identified the potential predictors of non-response by conducting chi-squared tests (tests of equality of proportions) on all key variables in our main analysis (i.e., mental health variables for parents and children and the sociodemographic controls). To adjust for non-response, we then calculated inverse probability weights following a two-step procedure, as outlined by Bartlett.^41^ First, we estimated a logit regression model, regressing the probability of being fully observed on the variables identified to influence missingness. Second, we calculated the inverse of the predicted values from these logit models and then used them as the probability weights in the full estimation sample, for both single and partnered parents. We have provided the weighted results as a robustness check to the rank-based model specifications.

It should be noted that, as we were using an unbalanced panel, sample attrition may affect the generalizability of its findings to the U.K. population. To test for attrition bias, we used the test proposed by Verbeek and Nijman^42^ with two test variables: (*a*) how many waves the adolescent was present in and (*b*) if the adolescent was present in the next wave. We regressed these test variables together with a full set of sociodemographic controls on adolescent mental health, using the rank-based model specification described above.

## Results

### Descriptive Characteristics

Table 1 below summarizes the pooled sample characteristics, split by marital status. As measured by SDQ, adolescent mental health was equal to 28.70 in single-mother households and to 29.80 in dual-parent households (with higher values indicating better mental health). As measured by GHQ-12, parental mental health was equal to 23.20 in single-parent households and 25.10 in dual-parent households (again, where higher values indicate better mental health). Approximately half of the adolescent sample were female.

**Table 1.** Descriptive Characteristics of the Pooled Sample.

	Single-mother		Dual-parent	
	Mean (SD)*	Count**	Mean (SD)	Count
***Adolescent characteristics***				
Total SDQ	28.70 (6.06)	1,419	29.80 (5.55)	5,383
Female	0.51 (0.50)	5,216	0.50 (0.50)	21,321
Age	11.30 (2.93)	5,220	10.60 (3.20)	21,321
Non-white	0.23 (0.42)	5,220	0.21 (0.41)	21,292
***Parental characteristics***				
GHQ-12	23.20 (6.35)	4,829	25.10 (4.02)	16,597
Age	40.20 (6.82)	5,220	42.20 (5.85)	21,321
With degree	0.35 (0.48)	5,188	0.61 (0.49)	21,040
***Region of residence***				
London	0.17 (0.38)	5,215	0.12 (0.33)	21,309
North East and West	0.14 (0.35)	5,215	0.14 (0.35)	21,309
Midlands	0.25 (0.43)	5,215	0.25 (0.43)	21,309
East	0.08 (0.27)	5,215	0.09 (0.29)	21,309
South	0.17 (0.38)	5,215	0.20 (0.40)	21,309
Wales	0.06 (0.24)	5,215	0.05 (0.22)	21,309
Scotland	0.08 (0.28)	5,215	0.08 (0.27)	21,309
Northern Ireland	0.05 (0.22)	5,215	0.06 (0.24)	21,309
***Economic characteristics***				
Monthly benefit income (£)	782.00	5,220	274.70 (313.40)	21,321
Benefit loss (£)	245.00	5,220	128.40 (195.90)	21,321
Household income (AHC) (£)	1379.70	5,220	2067.10 (2237.8)	21,321

*Decimal values indicate proportions

**”Count” indicates the total number of observations available for the variable.

The average age of parents in single-parent households was equal to 40.20 years whereas for single-parent households the age was 42.20 years. The proportion of adults with a degree was 35 percent in single-parent households and 61 percent in dual-parent households. The relatively young age of adolescents (approximately 11 years old) reflects the fact that we observe children/adolescents because their parents join the survey (and therefore their key demographic characteristics, including age and sex). Their mental health outcomes, however, are only available between ages 10–15, when they complete the youth questionnaire. When restricted to this subsample, the average age was approximately 12.50 years.

The most common region of residence was the Midlands for both groups (accounting for approximately 25% of observations), followed by the South (with 17% of single mothers and 20% of partnered parents living in this region).

Monthly equivalized household income (AHC) was equal to £1,398 in single-parent households and £2,067 in dual-parent households. In single-parent households, benefit income (equal to £782) accounted for over half of household income (56%). In dual-parent households, on the other hand, benefit income constituted 13 percent (equal to £275). Similarly, benefit losses were equal to £245 in single-parent households but only £128 in dual-parent households. These patterns are consistent with national statistics and distributional analyses of estimated benefit losses.^7,43^

### Missing Data

Out of all available variables in the analysis, the key missing variable was GHQ-12, absent from 5,115 out of 26,541 observations (19%). The availability of SDQ only in odd waves also significantly reduced the available number of observations; however, among the waves where SDQ was available, it was missing in only 1.4 percent of observations (98 out of 6,900).

To assess the predictors of item non-response, we conducted a series of chi-squared tests and found the following variables to influence non-response: adolescent age, ethnicity, parental age, and number of children in the household (for dual parents). For single mothers, the predictors of non-response included: adolescent mental health, age, ethnicity, parental age, education, and the number of children in the household. We therefore used these variables in the calculation of inverse probability weights and have provide the weighted results as a robustness check for the rank-based models. The chi-squared test results are presented in the Supplementary Material (Table 3S).

To test for mental health-related attrition bias, we used the test by Verbeek and Nijman.^42^ As illustrated in the Supplementary Material (Table 4S), the null hypothesis of random non-response from the Wald test cannot be rejected for either single- or dual-parent households. Therefore, we could assume that non-response would not bias the results.

### Regression Results

Table 2 illustrates the regression results for the intergenerational associations, as represented by the coefficients of percentile ranks of GHQ-12 (ranging 1–100). Model 1 represents the unadjusted association. Model 2 controls for adolescent sociodemographic characteristics (age, age squared, sex, and ethnicity) and Model 3 additionally controls for parental sociodemographic characteristics (age, age squared, education, number of children in the household, and region) and is the preferred baseline specification. Model 4 includes all controls used in Model 3 and also includes monthly household benefit losses to investigate their impact on intergenerational associations.

**Table 2.** Rank-Based Intergenerational Associations.

	Single-mother				Dual-parent			
	Model 1	Model 2	Model 3	Model 4	Model 1	Model 2	Model 3	Model 4
GHQ-12 rank	0.145***	0.144***	0.147***	0.145***	0.124***	0.120***	0.117***	0.110***
	(0.034)	(0.034)	(0.035)	(0.035)	(0.020)	(0.020)	(0.020)	(0.020)
***Adolescent characteristics***								
Age		−2.067	−1.362	−1.070		−2.190	−1.563	−1.632
		(4.516)	(4.527)	(4.496)		(2.361)	(2.370)	(2.366)
Age squared		0.134	0.096	0.081		0.092	0.059	0.063
		(0.194)	(0.195)	(0.194)		(0.104)	(0.105)	(0.105)
Sex (= female)		2.701	2.458	2.526		−0.947	−1.127	−1.050
		(1.773)	(1.749)	(1.750)		(1.074)	(1.072)	(1.070)
Ethnicity (= non-white)		5.855**	4.525	4.841*		5.246***	5.980***	6.653***
		(2.362)	(2.798)	(2.770)		(1.504)	(1.733)	(1.743)
***Parental characteristics***								
Age			1.921	1.911			3.859***	3.501***
			(1.552)	(1.557)			(1.160)	(1.169)
Age squared			−0.023	−0.023			−0.043***	−0.039***
			(0.019)	(0.019)			(0.013)	(0.013)
At least one parent with degree			−2.927	−3.118			3.846***	3.223***
			(2.077)	(2.072)			(1.202)	(1.218)
Number of children			−1.706	−1.682			−0.444	−0.037
			(1.899)	(1.865)			(0.821)	(0.839)
***Region (ref. London)***								
North East and West			1.362	1.207			6.076**	5.911**
			(4.266)	(4.262)			(2.526)	(2.524)
Midlands			−5.175	−5.303			2.403	2.304
			(3.643)	(3.644)			(2.290)	(2.294)
East			−6.776	−7.022			1.537	1.329
			(4.605)	(4.634)			(2.677)	(2.675)
South			−9.645**	−9.685**			2.880	2.760
			(4.308)	(4.283)			(2.333)	(2.337)
Wales			0.041	−0.325			8.473***	8.444***
			(5.231)	(5.217)			(3.155)	(3.141)
Scotland			−4.059	−4.402			4.879*	4.632*
			(4.478)	(4.501)			(2.700)	(2.709)
Northern Ireland			0.291	0.115			2.838	2.898
			(5.051)	(5.058)			(2.975)	(2.981)
**Log benefit loss**				−1.285				−1.288***
				(1.059)				(0.474)
Constant	42.724***	45.058*	11.594	17.448	43.878***	56.467***	−36.922	−23.631
	(2.078)	(26.169)	(41.302)	(41.571)	(1.209)	(13.155)	(28.053)	(28.400)
Observations	1348	1348	1340	1340	4278	4275	4202	4202
*R* ^2^	0.021	0.035	0.059	0.061	0.016	0.021	0.035	0.038

Model 1 includes no controls. Model 2 controls for adolescent characteristics (age, age squared, sex, and ethnicity). Model 3 additionally controls for parental characteristics (age, age squared, education, number of children, and region). Model 4 additionally includes monthly household benefit loss. Standard errors in parentheses (* *P* < .10, ** *P* < .05, *** *P* < .01).

The unadjusted (Model 1) results suggest that intergenerational correlation is higher for single- compared to dual-parent households (0.145 versus 0.124). Both correlations are highly statistically significant (*P* < .01). Adjusting for adolescent and parental sociodemographic characteristics has only reduced the intergenerational correlations for dual-parent households (from 0.124 to 0.117). The inclusion of benefit losses has reduced the intergenerational correlation for dual-parent households by 6 percent (from 0.117 to 0.110), whereas the correlation for single-mother households has only been reduced by 1 percent (from 0.147 to 0.145). Additionally, the results suggest that, while the independent effect of log benefit losses is non-significant for adolescents in single-mother households, it is negative in dual-parent households, whereby for every 10 percent decrease in benefits, SDQ falls by 0.05 percentiles^c^—a small but a statistically significant decrease in adolescent mental health.

For comparison, Table 5S in the Supplementary Material shows unit-based regression results whereby instead of mental health ranks, we use standardized averaged mental health unit measures for both parents and children. We can see that, compared to the regressions using percentile ranks, the unit-based regression coefficients have increased for dual-parent households and decreased slightly for single-mother households. Specifically, the coefficient for dual-parent households increased from 0.117 (Table 2, Model 3) to 0.182 (Supplementary Material: Table 5S, Model 3). The coefficient for single-mother households, on the other hand, has decreased slightly from 0.147 (Table 2, Model 3) to 0.123 (Supplementary Material: Table 5S, Model 3). Consistent with the results of rank-based models, the independent effect of benefit losses is negative and statistically significant only for dual-parent households.

Finally, the additional results displayed in the Supplementary Material (Table 6S) show that the inclusion of inverse probability weights used to control for missing data in general marginally decreased the levels of intergenerational correlation for single mothers (e.g., from 0.147 to 0.119 in Model 3 and from 0.145 to 0.121 in Model 4), therefore suggesting that the associations presented in this section may be considered an upper bound of the true estimate. For dual parents, the results remain very similar.

## Discussion

In this study, we investigated the strength of intergenerational correlations between parental and adolescent mental health, and the degree to which decreases in benefits contribute to this association. We found that there are sizable and statistically significant rank-based intergenerational correlations in mental health for both single- and dual-parent households, of approximately 0.15 and 0.12, respectively. This means that one percentile decrease in parental mental health is associated with decreases in mental health of their adolescent children of approximately 0.15 and 0.12 percentiles for single- and dual-parent households, respectively.

We also estimated unit-based associations and, while we found slightly lower correlations for single-mother households (0.12 versus 0.15 in unit- versus rank-based models), the intergenerational correlations for dual-parent households were considerably higher than the rank-based measures (equal to 0.12 versus 0.18 for the unit-based measure). This is in line with a recent, United Kingdom-based study by Bencsik and colleagues,^24^ who also estimated both rank- and unit-based coefficients for intergenerational transmission of mental health (but using the SF-12 measure) and found a unit-based estimate of 0.22, which was higher than the rank-based measure of 0.20. Most other previous literature on intergenerational transmission of mental health focused on unit-based measures and has similarly identified correlations ranging between 0.12 to 0.20,^18,22,23^ thus corroborating these findings.

In the (preferred) rank-based specifications, higher intergenerational correlations were found for single-mother as opposed to dual-parent households. Few previous studies have considered differences in intergenerational correlations in mental health between single- and dual-parent households. However, studies focusing on economic mobility, such as Chetty and colleagues,^40^ have found that there is lower mobility (thus higher correlation) in U.S. communities with high percentages of single mothers, in agreement with our findings for mental health.

We found that benefit losses explain a small proportion of intergenerational correlations for both single-mother and dual-parent households (1% and 6% respectively). These findings broadly reflect those in the study by Brown,^22^ who found no effect of changing policy environment on intergenerational correlations in mental health of either single- or dual-parent households the United Kingdom.

We also found that, when controlling for adolescent and parental characteristics (including parental mental health), benefit losses negatively contribute to the mental health of adolescents, though only in dual-parent households. Such findings are inconsistent with the “Parental Stress” theory—which would predict that benefit losses affect the mental health of adolescents primarily by affecting the mental health of their parents, suggesting that alternative explanations, such as the “Parental Investment” theory, may better explain our results.

Overall, our results are broadly consistent with the findings from a recent systematic review^25^ on the effects of social security reforms on mental health, which found that contractionary social security policies (i.e., those characterized by benefit cuts and/or eligibility) tend to be associated with decreases in mental health of both adults and children/adolescents. However, the subgroup-specific finding suggesting that the effect of benefit losses is negative and statistically significant for dual-parent but not for single-mother households is inconsistent with this research (which has found that such reforms tend to more adversely affect single- as opposed to dual-parent households). This inconsistency may have arisen due to the differences in the sample sizes between the two groups. The available sample sizes for dual-parent households were almost three time greater than those for single-mother households, thus leading to greater statistical power to detect significant differences in the former group. Nevertheless, there could be other potential explanations, making this inconsistency an important issue that warrants future investigation.

### Strengths and Limitations

To the best of our knowledge, this is the first analysis of the impacts of social security benefit decreases on the intergenerational transmission of mental health and inequalities in the United Kingdom. The analysis was conducted with a long-term panel study that allowed us to control for a wide range of confounders. We estimated both rank- and unit-based intergenerational associations, which gives a more complete picture of intergenerational mental health mobility in the United Kingdom. In addition, evidence shows that that rank-based measures are much less sensitive to specifications of the model and to attenuation and life-cycle bias^36,40^ and are therefore more robust compared to unit-based measures, which are still largely used by previous studies in this area. To further reduce the possibility of measurement error, we used time-averages of all variables for both parents and children, which helps ensure that our estimates are not attenuated by temporary shocks to mental health or errors in reporting. The availability of a long-term panel data also meant that we could calculate relatively long-term time averages for the parents (over 4.5 years on average), which is a recommended time frame for a reliable measure.^37^

Using a long-term panel data has its limitations, however. As we were using an unbalanced panel, whereby not all respondents appear in all waves, sample attrition may affect the generalizability of our findings to the U.K. population. To address the possibility of attrition bias, we conducted the Verbeek-Nijman^42^ attrition tests and found that mental health-related attrition bias was unlikely to affect the results. A related issue is non-response bias, whereby respondents may remain in the survey but not answer all questions relating to the key variables in our study, meaning that we are unable to use their data in the analysis. To address this issue, we calculated inverse probability weights based on key variables related to non-response and found that weighted results are slightly lower for single mothers and remain unchanged for partnered parents, suggesting that our estimates represent the upper bounds of intergenerational correlation in mental health for single mothers.

Another important limitation relating to measurement is that we relied on self-reported measures of mental health in this study, as opposed to clinical diagnoses, which are less prone to such errors. However, both GHQ-12 and SDQ have been widely used in longitudinal studies and are well validated measures in the U.K. population.^44,45^

A related limitation regarding measurement of mental health is that we utilized different measures for parental and adolescent mental health. While both measures are well validated and capture aspects of mental health such as concentration, worry, happiness, depression, and confidence, they are not identical. This may have led to an underestimation of the intergenerational associations.

Finally, a key limitation of this study is that the results may suffer from endogeneity bias and it is therefore not possible to establish causal relationships. We attempted to minimize this limitation by controlling for key observable characteristics affecting intergenerational correlations in mental health; however, low explanatory power of the econometric models meant that a number of unobserved characteristics remained unaccounted for. Thus, these results should be interpreted as associations and not as causal relationships.

### Future Research

Our findings point to several potential avenues for future research. First, the associational nature of the results provides a first step to further investigate the mechanisms relating social security benefit changes and intergenerational transmission of mental health. For example, using dynamic panel data models and structural models could help control for endogeneity bias and establish causal relationships and therefore help to better understand the role of social security benefit changes in determining the intergenerational transmission of mental health. Second, it is important to further investigate the potential mechanisms by which decreases in benefits affect the mental health of adolescents in dual-parent households (e.g., parental investment-related factors). Finally, when estimating intergenerational correlations, future researchers could utilize administrative health records with clinical diagnoses to verify estimates that are based on self-reports, ideally, using the same measures for both parents and their offspring.

## Conclusion

Our results suggest that there are significant intergenerational associations in mental health for both single- and dual-parent households, with the relationship being stronger for single-mother households. While benefit losses have negative effects on adolescent mental health in dual-parent households, they explain a small proportion of the intergenerational associations in mental health for both types of households. This indicates that benefit losses may adversely affect adolescent mental health in dual-parent households largely independently of their parents, therefore suggesting that alternative mechanisms should be explored. Understanding such negative effects is particularly important for designing future social security policies to help ensure that they protect, or at least do not undermine, the mental health of our future generations.

## Supplemental Material

sj-docx-1-joh-10.1177_27551938231185948 - Supplemental material for Understanding the Relationship Between Decreases in Social Security Benefits and Intergenerational Inequalities in Mental Health Click here for additional data file. Supplemental material, sj-docx-1-joh-10.1177_27551938231185948 for Understanding the Relationship Between Decreases in Social Security Benefits and Intergenerational Inequalities in Mental Health by Julija Simpson, Clare Bambra and Heather Brown in International Journal of Social Determinants of Health and Health Services

## Notes

a. Please note: Due to the low numbers of single fathers available in the survey (< 4% of total sample), we focused solely on single mothers.

b. For example, the study by Garber and Cole^33^ investigated the relationship between maternal and adolescent depression using different measures of depression. For mothers, the measure was the Brief Symptom Inventory, whereas for adolescents the measure was modified Children's Depression Rating Scale.

c. Calculated as: log(1.10)×(−1.288) = −0.05
