# Supplementary material for: Understanding the Relationship Between Decreases in Social Security Benefits and Intergenerational Inequalities in Mental Health
Source: Int J Soc Determinants Health Health Serv. 2023 Jul 4;53(4):403–13. doi: 10.1177/27551938231185948 (PMC10631259; doi:10.1177/27551938231185948)
Supplement: sj-docx-1-joh-10.1177_27551938231185948 - Supplemental material for Understanding the Relationship Between Decreases in Social Security Benefits and Intergenerational Inequalities in Mental Health [file sj-docx-1-joh-10.1177_27551938231185948.docx]

1. **Comparison of GHQ-12 and SDQ questionnaires**

This part of supplementary material illustrates the comparison between the GHQ-12 and SDQ mental health measures. For completeness, first, all GHQ-12 items are presented. Second, all SDQ scoring items are presented. Finally, a side-by-side comparison of the relevant items is provided.

**GHQ-12 Questions^[[1]](#footnote-1)^**

Each item can be rated on a four-item Likert scale (e.g., better than usual, same as usual, less than usual, much less than usual – depending on the item).

Have you recently:

1. Been able to concentrate on what you are doing?
2. Lost much sleep over worry?
3. Felt you were playing a useful part in things?
4. Felt capable of making decisions about things?
5. Felt constantly under strain?
6. Felt you couldn’t overcome your difficulties?
7. Been able to enjoy your day to day activities?
8. Been able to face up your problems?
9. Been feeling unhappy or depressed?
10. Been losing confidence in yourself?
11. Been thinking of yourself as a worthless person?
12. Been feeling reasonably happy, all things considered?

**SDQ Items**^[[2]](#footnote-2)^ (that are used in the summary score 0-40 – this includes all sub-scales, except the ‘prosocial’).

Each item can be selected as either: Not True, Somewhat True, Certainly True:

1. I am restless. I cannot stay still for long.
2. I get a lot of headaches, stomach-aches or sickness.
3. I get very angry and often lose my temper.
4. I am usually on my own. I generally play alone or keep to myself.
5. I usually do as I am told.
6. I worry a lot.
7. I am constantly fidgeting or squirming.
8. I have one good friend or more.
9. I fight a lot. I can make other people do what I want.
10. I am often unhappy, down-hearted or tearful.
11. Other people my age generally like me.
12. I am easily distracted, I find it difficult to concentrate.
13. I am nervous in new situations. I easily lose confidence.
14. I am often accused of lying or cheating.
15. Other children or young people pick on me or bully me.
16. I think before I do things.
17. I take things that are not mine from home, school, or elsewhere.
18. I get on better with adults than with people my own age.
19. I have many fears. I am easily scared.
20. I finish the work I'm doing. My attention is good.

**Comparison of GHQ-12 and SDQ items**

Table 1S below illustrates five GHQ-12 items, which have an equivalent for the SDQ items, making these two measures comparable for the intergenerational correlations analysis.

*Table 1S: Side-by-side comparison of alternative GHQ-12 and SDQ items*

| **GHQ-12 item** | **Similar/Equivalent SDQ item** |
| --- | --- |
| Been able to concentrate on what you are doing. | I am easily distracted. I find it difficult to concentrate. |
| Lost much sleep over worry. | I worry a lot. |
| Been feeling unhappy or depressed. | I am often unhappy, downhearted, or tearful. |
| Been losing confidence in yourself. | I am nervous in new situations. I easily lose confidence. |
| Been feeling reasonably happy, all things considered. | I am often unhappy, downhearted, or tearful. |

1. **Definitions of variables used in the analysis**

Table 2S Definitions of variables used in the analysis

| **Variable** | **Definition** | **Original Variable(s)** |
| --- | --- | --- |
|  |  |  |
| ***Mental health variables*** |  |  |
| *Z_SDQ_AVERAGE* | Reverse coded, standardised total SDQ, averaged across waves for each adolescent | YPSDQTD_DV |
| *Z_GHQ_M_AVERAGE* | Reverse coded, standardised GHQ-12 averaged across waves for each mother | SCGHQ1_DV |
| *Z_GHQ_B_AVERAGE* | Reverse coded, standardised GHQ-12 averaged across waves for each partnered parent couple | SCGHQ1_DV |
| *RANK_KIDS_SINGLE* | Percentile rank (1-100) of average adolescent SDQ (single parent households) | YPSDQTD_DV |
| *RANK_KIDS_DUAL* | Percentile rank (1-100) of average adolescent SDQ (dual parent households) | YPSDQTD_DV |
| *RANK_SINGLE* | Percentile rank (1-100) of average maternal GHQ | SCGHQ1_DV |
| *RANK_DUAL* | Percentile rank (1-100) of average parental GHQ (for partnered parents) | SCGHQ1_DV |
| ***Adolescent characteristics*** |  |  |
| *AVERAGE_AGE_K* | Average age of the adolescent | AGE |
| *AVERAGE_AGE_SQ_K* | Average age of the adolescent squared | AGE |
| *SEX_K* | Sex of the adolescent (0=Male; 1=Female) | SEX |
| *NON-WHITE* | Ethnicity of the adolescent (0=White; 1=Non-White) | ETHN_DV |
| ***Parental characteristics*** |  |  |
| *SINGLE_M* | Partnership status:  0=Partnered (married/ cohabiting.  1=Single (never married, separated, divorced, widowed) | MASTAT_DV |
| *AVERAGE_AGE_M* | Average maternal age | AGE_DV |
| *AVERAGE_AGE_SQ_M* | Average maternal age squared | AGE_DV |
| *AVERAGE_AGE_B* | Average parental age | AGE_DV |
| *AVERAGE_AGE_SQ_B* | Average parental age squared | AGE_DV |
| *EVER_HAD_A_DEGREE_M* | Mother has a degree | HIQUAL_DV |
|  |  |  |
| *EVER_HAD_A_DEGREE_B* | At least one parent has a degree | HIQUAL_DV |
|  |  |  |
| *MEAN_NUMBER_OF_CHILDREN* | Number of children aged 0-15 in the household | NCH02_DV; NCH34_DV; NCH511_DV; NCH1215_DV |
| *REGION*^[[3]](#footnote-3)^ | Categorical variable indicating region of residence  1=London  2=North East and West  3=Midlands  4=East | GOR_DV |
|  | 5=South |  |
|  | 6=Wales  7=Scotland  8=Northern Ireland |  |
|  |  |  |
| *MEAN_LOG_BENEFIT_LOSS* | Log average benefit loss of the HH between t-1 and t – two  subsequent waves (inflation adjusted and equivalised) | FIHHMNSBEN_DV |
| *MEAN_JOB_HOURS_M* | Average weekly maternal job hours | JBHRS |
| *MEAN_JOB_HOURS_B* | Average weekly job hours between mothers and fathers | JBHRS |

1. **Chi-Squared Test Results**

To conduct the chi-squared tests for investigating patterns of non-response, we first needed to convert our continuous variables into categorical, as explained below.

For SDQ, we created a binary variable indicating emotional–behavioral problems vs no emotional–behavioral problems, based on a validated cut-off point of >16 = emotional–behavioral problems, <16 no problems (Goodman, 1997). For GHQ-12, we used the caseness variables available in the survey, the established cut off for presence of psychological distress is the score of 4 or more on the caseness scale (Health survey England, 2014).

Parental age was converted into four categories: 1 = Less than 30 years old; 2 = Between 30-40; 3 = Between 41–50; 4 = Older than 50.

The number of children in the household variable was split into two categories 1 = one to two children; 2 = three or more children.

The other variables remained unchanged from their original specification.

The chi-squared test results are illustrated in Table C1 below. The variables that were statistically significant at 5 percent level were included in the estimation for non-response weights.

*Table 3S Chi-squared test results*

| **Chi-squared test*** |  | **Single mothers** | **Statistically significant at 5% level (yes/no)** | **Dual parents** | **Statistically significant at 5% level (yes/no)** |
| --- | --- | --- | --- | --- | --- |
| Total SDQ (binary) | Pearson Chi2(1) | 7.7301 |  | 0.0979 |  |
|  | P>Chi2> | 0.005 | Yes | 0.754 | No |
| Parental psychological distress (GHQ binary) | Pearson Chi2(1) | 0.6652 |  | 0.8258 |  |
| **Chi-squared test*** |  | **Single mothers** | **Statistically significant at 5% level (yes/no)** | **Dual parents** | **Statistically significant at 5% level (yes/no)** |
|  | P>Chi2> | 0.415 | No | 0.662 | No |
| Adolescent age | Pearson Chi2(1) | 44.2433 |  | 30.0206 |  |
|  | P>Chi2> | 0.000 | Yes | 0.000 | Yes |
| Adolescent sex | Pearson Chi2(1) | 0.0994 |  | 0.0502 |  |
|  | P>Chi2> | 0.753 | No | 0.823 | No |
| Adolescent non-White | Pearson Chi2(1) | 7.2467 |  | 234.7260 |  |
|  | P>Chi2> | 0.007 | Yes | 0.000 | Yes |
| Parental age (categorical) | Pearson Chi2(1) | 275.8212 |  | 718.6544 |  |
|  | P>Chi2> | 0.000 | Yes | 0.000 | Yes |
| A parent with a degree | Pearson Chi2(1) | 0.7345 |  | 2.3230 |  |
|  | P>Chi2> | 0.391 | Yes | 0.127 | No |
| Number of children | Pearson Chi2(1) | 15.7548 |  | 62.7640 |  |
|  | P>Chi2> | 0.000 | Yes | 0.000 | Yes |
| Region | Pearson Chi2(1) | 85.1260 |  | 8.6499 |  |
|  | P>Chi2> | 0.000 | Yes | 0.279 | No |

*Chi-squared tests for the listed variables were conducted for both single- and dual-parent households given the separately estimated models in the main analysis. Inverse probability weights for these two groups were thus also calculated separately.

**References:**

Goodman, R. (1997) ‘The Strengths and Difficulties Questionnaire: a research note’, *Journal of child psychology and psychiatry*, 38(5), pp. 581-586.

Health Survey England (2014) *Mental health problems*. Available at: <http://healthsurvey.hscic.gov.uk/support-guidance/public-health/health-survey-for-england-2014/mental-health-problems.aspx> (Retrieved: 22 May 2022).

1. **Attrition test results**

Table 4S Results of the Verbeek-Nijman (1992) attrition test

| **Wald test for attrition** |  | **Single mothers** | **Dual parents** |
| --- | --- | --- | --- |
| Number of waves individual is present | Chi2(1) | 2.47 | 0.96 |
|  | P>Chi2> | 0.1161 | 0.3269 |
| If individual is present in next wave | Chi2(1) | 0.60 | 0.15 |
|  | P>Chi2> | 0.4392 | 0.9652 |

For both single mothers and dual parents, the null hypothesis of no non-random attrition cannot be rejected.

1. **Linear (unit-based) intergenerational associations**

*Table 5S Unit-based intergenerational associations*

|  | Single mother | | | | Dual parent | | | |
| --- | --- | --- | --- | --- | --- | --- | --- | --- |
|  | Model 1 | Model 2 | Model 3 | Model 4 | Model 1 | Model 2 | Model 3 | Model 4 |
| Standardized GHQ | 0.115^***^ | 0.118^***^ | 0.123^***^ | 0.122^***^ | 0.192^***^ | 0.188^***^ | 0.182^***^ | 0.171^***^ |
|  | (0.036) | (0.036) | (0.037) | (0.037) | (0.031) | (0.031) | (0.031) | (0.031) |
| ***Adolescent characteristics*** |  |  |  |  |  |  |  |  |
| Age |  | -0.036 | -0.003 | 0.007 |  | -0.022 | -0.002 | -0.005 |
|  |  | (0.164) | (0.166) | (0.165) |  | (0.075) | (0.075) | (0.075) |
|  |  |  |  |  |  |  |  |  |
| Age squared |  | 0.003 | 0.002 | 0.001 |  | 0.001 | -0.000 | -0.000 |
|  |  | (0.007) | (0.007) | (0.007) |  | (0.003) | (0.003) | (0.003) |
|  |  |  |  |  |  |  |  |  |
| Sex (=female) |  | 0.109^*^ | 0.100 | 0.102 |  | -0.019 | -0.027 | -0.024 |
|  |  | (0.063) | (0.062) | (0.062) |  | (0.034) | (0.033) | (0.033) |
|  |  |  |  |  |  |  |  |  |
| Ethnicity (=non-White) |  | 0.235^***^ | 0.177^*^ | 0.187^**^ |  | 0.159^***^ | 0.176^***^ | 0.200^***^ |
|  |  | (0.079) | (0.093) | (0.092) |  | (0.046) | (0.056) | (0.056) |
| ***Parental characteristics*** |  |  |  |  |  |  |  |  |
| Age |  |  | 0.071 | 0.071 |  |  | 0.130^***^ | 0.117^***^ |
|  |  |  | (0.054) | (0.055) |  |  | (0.038) | (0.038) |
|  |  |  |  |  |  |  |  |  |
| Age squared |  |  | -0.001 | -0.001 |  |  | -0.001^***^ | -0.001^***^ |
|  |  |  | (0.001) | (0.001) |  |  | (0.000) | (0.000) |
|  |  |  |  |  |  |  |  |  |
| Has a degree |  |  | -0.093 | -0.099 |  |  | 0.120^***^ | 0.098^**^ |
|  |  |  | (0.073) | (0.073) |  |  | (0.038) | (0.039) |
|  |  |  |  |  |  |  |  |  |
| Number of children |  |  | -0.062 | -0.061 |  |  | -0.018 | -0.004 |
|  |  |  | (0.064) | (0.063) |  |  | (0.027) | (0.027) |
| ***Region (ref. London)*** |  |  |  |  |  |  |  |  |
| North East and West |  |  | 0.041 | 0.036 |  |  | 0.156^*^ | 0.149^*^ |
|  |  |  | (0.140) | (0.140) |  |  | (0.080) | (0.080) |
|  |  |  |  |  |  |  |  |  |
| Midlands |  |  | -0.202^*^ | -0.206^*^ |  |  | 0.058 | 0.054 |
|  |  |  | (0.121) | (0.121) |  |  | (0.071) | (0.071) |
|  |  |  |  |  |  |  |  |  |
| East |  |  | -0.296^*^ | -0.304^*^ |  |  | 0.027 | 0.019 |
|  |  |  | (0.167) | (0.168) |  |  | (0.081) | (0.081) |
|  |  |  |  |  |  |  |  |  |
| South |  |  | -0.383^***^ | -0.385^***^ |  |  | 0.069 | 0.064 |
|  |  |  | (0.146) | (0.145) |  |  | (0.072) | (0.073) |
|  |  |  |  |  |  |  |  |  |
| Wales |  |  | 0.009 | -0.002 |  |  | 0.236^**^ | 0.234^**^ |
|  |  |  | (0.170) | (0.170) |  |  | (0.099) | (0.099) |
|  |  |  |  |  |  |  |  |  |
| Scotland |  |  | -0.156 | -0.167 |  |  | 0.126 | 0.117 |
|  |  |  | (0.149) | (0.150) |  |  | (0.082) | (0.082) |
|  |  |  |  |  |  |  |  |  |
| Northern Ireland |  |  | 0.008 | 0.002 |  |  | 0.070 | 0.072 |
|  |  |  | (0.172) | (0.172) |  |  | (0.092) | (0.093) |
|  |  |  |  |  |  |  |  |  |
| **Log benefit loss** |  |  |  | -0.040 |  |  |  | -0.046^***^ |
|  |  |  |  | (0.035) |  |  |  | (0.015) |
|  |  |  |  |  |  |  |  |  |
| Constant | -0.107^***^ | -0.287 | -1.584 | -1.404 | 0.065^***^ | 0.211 | -2.892^***^ | -2.430^***^ |
|  | (0.036) | (0.945) | (1.487) | (1.493) | (0.018) | (0.417) | (0.892) | (0.903) |
| Observations | 1348 | 1348 | 1340 | 1340 | 4278 | 4275 | 4202 | 4202 |
| *R*^2^ | 0.011 | 0.029 | 0.056 | 0.057 | 0.018 | 0.022 | 0.037 | 0.040 |

Model 1 includes no controls. Model 2 controls for adolescent characteristics (age, age squared, sex, and ethnicity). Model 3 additionally controls for parental characteristics (age, age squared, education, number of children, and region). Model 4 additionally includes monthly household benefit loss. Standard errors in parentheses (* *p* < 0.10, ** *p* < 0.05, *** *p* < 0.01).

1. **Inverse Probability Weighted Results**

*Table 6S Inverse probability weighted results*

|  | Single-mother | | | | Dual-parent | | | |
| --- | --- | --- | --- | --- | --- | --- | --- | --- |
|  | Model 1 | Model 2 | Model 3 | Model 4 | Model 1 | Model 2 | Model 3 | Model 4 |
| GHQ rank | 0.099 | 0.090 | 0.119^***^ | 0.121^***^ | 0.123^***^ | 0.117^***^ | 0.118^***^ | 0.113^***^ |
|  | (0.070) | (0.069) | (0.037) | (0.036) | (0.021) | (0.021) | (0.021) | (0.021) |
| ***Adolescent characteristics*** |  |  |  |  |  |  |  |  |
| Age |  | -4.725 | -2.118 | -1.769 |  | -0.243 | 0.156 | 0.116 |
|  |  | (5.762) | (5.030) | (4.942) |  | (2.526) | (2.515) | (2.513) |
|  |  |  |  |  |  |  |  |  |
| Age squared |  | 0.254 | 0.130 | 0.112 |  | 0.002 | -0.017 | -0.015 |
|  |  | (0.239) | (0.211) | (0.207) |  | (0.111) | (0.111) | (0.111) |
|  |  |  |  |  |  |  |  |  |
| Female |  | 2.375 | 2.536 | 2.606 |  | -0.659 | -0.942 | -0.867 |
|  |  | (1.875) | (1.746) | (1.742) |  | (1.104) | (1.105) | (1.104) |
|  |  |  |  |  |  |  |  |  |
| Non-White |  | 6.279^**^ | 2.847 | 3.353 |  | 5.087^***^ | 5.808^***^ | 6.377^***^ |
|  |  | (2.836) | (2.859) | (2.809) |  | (1.556) | (1.767) | (1.785) |
| ***Parental characteristics*** |  |  |  |  |  |  |  |  |
| Age |  |  | 2.458 | 2.415 |  |  | 3.196^***^ | 2.909^**^ |
|  |  |  | (1.639) | (1.634) |  |  | (1.167) | (1.179) |
|  |  |  |  |  |  |  |  |  |
| Age squared |  |  | -0.028 | -0.027 |  |  | -0.036^***^ | -0.033^**^ |
|  |  |  | (0.020) | (0.020) |  |  | (0.013) | (0.014) |
|  |  |  |  |  |  |  |  |  |
| Has a degree |  |  | -1.846 | -2.041 |  |  | 3.925^***^ | 3.377^***^ |
|  |  |  | (2.165) | (2.155) |  |  | (1.241) | (1.267) |
|  |  |  |  |  |  |  |  |  |
| Number of children |  |  | 1.184 | 1.127 |  |  | -0.250 | 0.084 |
|  |  |  | (1.435) | (1.392) |  |  | (0.910) | (0.931) |
| ***Region (ref. London)*** |  |  |  |  |  |  |  |  |
|  |  |  |  |  |  |  |  |  |
| North East and West |  |  | -5.795 | -5.974 |  |  | 5.987^**^ | 5.842^**^ |
|  |  |  | (4.704) | (4.662) |  |  | (2.592) | (2.598) |
|  |  |  |  |  |  |  |  |  |
| Midlands |  |  | -9.562^***^ | -9.675^***^ |  |  | 3.239 | 3.166 |
|  |  |  | (3.571) | (3.550) |  |  | (2.372) | (2.382) |
|  |  |  |  |  |  |  |  |  |
| East |  |  | -10.543^**^ | -10.674^**^ |  |  | 1.330 | 1.154 |
|  |  |  | (4.891) | (4.872) |  |  | (2.722) | (2.725) |
|  |  |  |  |  |  |  |  |  |
| South |  |  | -17.090^***^ | -17.049^***^ |  |  | 3.165 | 3.056 |
|  |  |  | (4.424) | (4.372) |  |  | (2.423) | (2.433) |
|  |  |  |  |  |  |  |  |  |
| Wales |  |  | -4.020 | -4.430 |  |  | 9.474^***^ | 9.397^***^ |
|  |  |  | (5.257) | (5.214) |  |  | (3.240) | (3.237) |
|  |  |  |  |  |  |  |  |  |
| Scotland |  |  | -8.278^*^ | -8.758^*^ |  |  | 4.984^*^ | 4.785^*^ |
|  |  |  | (4.650) | (4.698) |  |  | (2.800) | (2.809) |
|  |  |  |  |  |  |  |  |  |
| Northern Ireland |  |  | -2.106 | -2.267 |  |  | 3.834 | 3.845 |
|  |  |  | (4.763) | (4.749) |  |  | (3.072) | (3.079) |
|  |  |  |  |  |  |  |  |  |
| **Log benefit loss** |  |  |  | -1.772 |  |  |  | -1.045^**^ |
|  |  |  |  | (1.178) |  |  |  | (0.487) |
|  |  |  |  |  |  |  |  |  |
| Constant | 45.303^***^ | 62.450^*^ | 0.845 | 9.823 | 44.494^***^ | 46.424^***^ | -31.414 | -20.830 |
|  | (4.751) | (37.857) | (43.498) | (43.836) | (1.239) | (14.122) | (28.172) | (28.595) |
| Observations | 1348 | 1348 | 1340 | 1340 | 4273 | 4273 | 4202 | 4202 |
| *R^2^* | 0.010 | 0.026 | 0.073 | 0.076 | 0.016 | 0.022 | 0.035 | 0.037 |

Model 1 includes no controls. Model 2 controls for adolescent characteristics (age, age squared, sex and ethnicity). Model 3 additionally controls for parental characteristics (age, age squared, education, number of children and region). Model 4 additionally includes monthly household benefit loss. Standard errors in parentheses (* p < 0.10, ** p < 0.05, *** p < 0.01)

1. Source: Goldberg DP, Williams P: *A User’s Guide to the General Health Questionnaire.* Windsor: NFER-Nelson; 1988. [↑](#footnote-ref-1)
2. Source: Youth in Mind (2022) *SDQ*. Available at: https://www.sdqinfo.org/ (Accessed: 22/06/2022). [↑](#footnote-ref-2)
3. Please note: This variable was mostly time invariant within individuals (i.e., remained the same in over 99.5% of observations) and therefore was not time averaged. [↑](#footnote-ref-3)
